# Opportunities and challenges of traditional Chinese medicine doctors in the era of artificial intelligence

**DOI:** 10.3389/fmed.2023.1336175

**Published:** 2024-01-11

**Authors:** Wenyu Li, Xiaolei Ge, Shuai Liu, Lili Xu, Xu Zhai, Linyong Yu

**Affiliations:** ^1^School of Marxism, Capital Normal University, Beijing, China; ^2^Wangjing Hospital of China Academy of Traditional Chinese Medicine, Beijing, China; ^3^Graduate School of Chinese Academy of Traditional Chinese Medicine, Beijing, China; ^4^China Academy of Chinese Medical Sciences, Beijing, China

**Keywords:** artificial intelligence, traditional Chinese medicine, Chinese medicine doctor, opportunities, challenges

## Abstract

With the exponential advancement of artificial intelligence (AI) technology, the realm of medicine is experiencing a paradigm shift, engendering a multitude of prospects and trials for healthcare practitioners, encompassing those devoted to the practice of traditional Chinese medicine (TCM). This study explores the evolving landscape for TCM practitioners in the AI era, emphasizing that while AI can be helpful, it cannot replace the role of TCM practitioners. It is paramount to underscore the intrinsic worth of human expertise, accentuating that artificial intelligence (AI) is merely an instrument. On the one hand, AI-enabled tools like intelligent symptom checkers, diagnostic assistance systems, and personalized treatment plans can augment TCM practitioners’ expertise and capacity, improving diagnosis accuracy and treatment efficacy. AI-empowered collaborations between Western medicine and TCM can strengthen holistic care. On the other hand, AI may disrupt conventional TCM workflow and doctor-patient relationships. Maintaining the humanistic spirit of TCM while embracing AI requires upholding professional ethics and establishing appropriate regulations. To leverage AI while retaining the essence of TCM, practitioners need to hone holistic analytical skills and see AI as complementary. By highlighting promising applications and potential risks of AI in TCM, this study provides strategic insights for stakeholders to promote the integrated development of AI and TCM for better patient outcomes. With proper implementation, AI can become a valuable assistant for TCM practitioners to elevate healthcare quality.

## Introduction

In recent years, significant progress has been made in artificial intelligence (AI), with the emergence of systems such as AlphaGo and ChatGPT generating widespread attention and anticipation (1, 2). The remarkable achievement of AlphaGo in defeating human experts in the game of Go was awe-inspiring (3). Furthermore, the rise of generative AI technologies like ChatGPT has stimulated boundless imagination regarding the prospects of general AI. These systems’ successful applications and rapid dissemination have propelled in-depth research on generative AI technologies worldwide (4).

AI has already demonstrated positive impacts in the healthcare domain and has provided new opportunities for achieving health equity, optimizing diagnostic processes, and effectively utilizing healthcare resources (5). AI technology has enhanced doctors’ efficiency, improved doctor-patient relationships, and prioritized patient-centered care. By leveraging complex combinations of big data, AI has established standardized data systems and facilitated medical education. This aligns with the principles of TCM and patient-centered medical ethics, emphasizing the harmony between humans and nature (6, 7). AI’s ability to process information efficiently allows doctors to allocate more time for patient communication, fostering a more compassionate doctor-patient relationship and reinforcing patient-centered care.

Notably, in the progress of AI technology, significant breakthroughs have been achieved in detecting coronary artery disease using deep learning algorithms. Researchers have successfully developed and validated a novel coronary artery disease detection method based on facial photos using deep learning algorithms. This method exhibits outstanding performance, with a sensitivity of 80% and specificity of 54%, surpassing traditional risk prediction models. This innovative research offers a potential new approach to screening for coronary artery disease, enabling more accurate diagnosis and prediction through facial feature analysis.

Moreover, researchers have extensively employed deep learning techniques in prescription analysis to extract valuable guidance for traditional Chinese medicine (TCM) research and clinical practice by exploring potential information within prescription data (8). Despite the remarkable achievements of AI in the healthcare field, various challenges and limitations persist. Key issues requiring urgent resolution include ensuring information accuracy, safeguarding data privacy, and addressing ethical and moral concerns. Future research efforts should address these challenges to ensure AI technology’s sustainable development and application (9). Therefore, AI is an emerging technology that involves complex relationships among patients, TCM practitioners, and non-living systems. Patients need to ensure their personal privacy and data security when receiving AI-assisted diagnosis and treatment. On the other hand, TCM practitioners need to uphold the core values of professional knowledge and compassionate care when applying AI. Finding a balance between these relationships requires further research and exploration. This paper presents the viewpoint and explores the current status and issues related to the topic.

### The application of AI in TCM diagnosis and treatment

During diagnosis, TCM employs multiparametric assessment, including inspection, auscultation and olfaction, inquisition, and palpation (MAP) to obtain informative cues for pattern differentiation and treatment. Data mining techniques can effectively analyze real-world prescribing patterns in TCM clinical practice. For example, one study (10) applied association rule learning to electronic medical records from an integrative Chinese-Western pharmacy, finding batched prescription rules and guidelines to optimize allocation across multiple intelligent devices. Regarding pulse diagnosis, research (11) has proposed a graph-based multichannel feature fusion method using two sensors to extract distinct features from the three pulse modalities, constructing sample graphs to aid diagnosis via graph convolutional networks. Experimental results demonstrated superior performance over other state-of-the-art approaches. One team (12) developed an intelligent tongue recognition system leveraging deep learning to rapidly and accurately identify pathological characteristics in tongue and facial diagnostics. The tongue diagnosis framework (13) incorporates YOLOv5s6, U-Net, and MobileNetV3 networks for tongue segmentation, boundary of regions, and feature classification of teeth marks, spots, and fissures. The system achieved classification accuracies of 93.33, 89.60, and 97.67%, respectively, representing a valuable reference for objective, intelligent tongue examination. Recent studies in other medical disciplines have indicated that the level of autonomy in applying AI in robotic surgery is currently low, and future assessments will focus on outcome-oriented processes (14). With AI-assisted, it can be assumed that these technologies can support physicians in other clinical disciplines to work better and faster. Within the scope of artificial intelligence, improving the accuracy of diagnostic techniques involves a comprehensive transformation from a technological perspective to the improvement of doctor-patient relationships (15). This comprehensive transformation is a technological advancement and a holistic development of the medical field under the guidance of artificial intelligence.

However, standardized mobile phone-based imaging can reduce environmental interference while expediting medical image analysis, providing a feasible approach for applying camera-captured images in this domain. Overall, AI-assisted techniques show promise for advancing data-driven insights and precision approaches across diverse areas of TCM evaluation.

In addition, research suggests that TCM is suitable for applying AI in medical diagnosis and treatment. TCM has a rich history of practice and has developed a complete theoretical system, providing excellent conditions for the application of AI (16). On the one hand, the extensive clinical experience accumulated over thousands of years has built a large-scale database, offering abundant learning samples for training AI models. On the other hand, TCM theory encompasses a complex interactive system that considers comprehensive etiology and pathogenesis, relying on the clinical decision-making experience accumulated by physicians. Furthermore, the application scenarios of TCM vary geographically and among practitioners, leading to many factors that can influence diagnostic outcomes, making it difficult to predict outcomes with certainty. However, these characteristics also make TCM suitable for the advantages of AI in handling large-scale data and multiple features, providing valuable references for AI learning.

Moreover, TCM diagnosis and treatment rely on physicians’ rich clinical judgment experience, which provides valuable reference examples for AI to learn and emulate (17). Additionally, TCM emphasizes the adjustment of treatment methods based on changes in the patient’s condition, which aligns with the progressive nature of scientific research. AI can further optimize diagnosis and treatment plans through data-driven approaches. The regional and practitioner variations in TCM allow AI to explore common patterns and regularities (18). With the development of biomedical technology, various assessment indicators in TCM are continuously being quantified, providing a more prosperous basis for the application of AI. In summary, the discourse system of TCM aligns well with the processing capabilities of AI, laying a solid foundation for further collaboration and communication.

### Opportunity

Traditional Chinese medicine embodies the valuable experience and innovative thinking accumulated over millennia in medical practice. As an evolving medical system, TCM has demonstrated underappreciated strengths across many health domains. Rapid artificial intelligence advancement provides new opportunities for TCM. AI excels at efficiently analyzing complex datasets, unprecedentedly facilitating the exploration and validation of TCM theories. AI also aids TCM modernization, including AI-assisted drug virtual screening and prediction of candidate drug absorption, distribution, metabolism, excretion, and toxicity (19). AI-assisted diagnostic systems achieved good accuracy for 187 common TCM patterns (20). In addition, AI empowers more personalized TCM treatments. By analyzing individual symptoms, medical history, and health metrics, AI models can recommend tailored herb formulas and acupoint prescriptions aligned with a patient’s TCM pattern differentiation (21). This supports the inherent individualized nature of TCM practice. AI also facilitates large-scale data mining to uncover new TCM knowledge from clinical experience (22). Machine learning models applied to herbal medicine databases have discovered novel herb combinations and network pharmacology relationships (23, 24). AI brings new prospects and powerful analytical tools supporting TCM research, clinical practice, and modern scientific verification of models. Thus, with deepening AI-TCM integration, TCM practitioners face new development opportunities. They can leverage AI to enhance training and participate in modern validation efforts. Digital platforms will provide an expanded stage for continuing TCM’s unique preventive and healthcare roles. When exploring TCM practitioner futures, its rich historical wisdom must be appreciated. TCM crystallizes invaluable experience from centuries of human medical practice, providing an invaluable knowledge repository. In summary, judicious AI-TCM integration has the potential to advance the appreciation and modernization of TCM while preserving its core holistic values.

However, AI can potentially reduce management costs, especially in healthcare systems that are outcome-driven and payment-based (25, 26). Through automated and intelligent technologies, AI can enhance the efficiency of TCM physicians, alleviating their workload and making their lives easier. For instance, AI automates tedious tasks such as document recording and report generation, saving doctors time and energy. However, responsible development of AI-TCM requires upholding ethics, preventing over-reliance on algorithms, and maintaining sound doctor-patient relationships. With prudent implementation, AI can become a valuable assistant for TCM practitioners to elevate healthcare quality in the modern era.

### Challenge

#### Lack of TCM humanistic care in artificial intelligence

“The first premise of all human history is undoubtedly the existence of living individuals” (27). Although AI has positively impacted various aspects of TCM research, it is essential to consider the human aspect of TCM services since the recipients of TCM care are human beings. Shared decision making (SDM), as an important approach to bridge the gap in evidence-based medical practice, places the “patient at the center” and encourages patients to participate in discussions about their diagnosis, treatment, and follow-up, thereby facilitating the development of personalized clinical decisions that best suit the patient’s needs (28, 29). The influence of AI on SDM remains unclear, and its application comes with various risks and challenges. Currently, AI cannot co-achieve individualized clinical decisions with patients in the TCM field. Overall, it is mainly manifested in several aspects. First, AI is a technology based on algorithms and data, unable to experience emotions or demonstrate empathy. AI cannot understand patients’ moods, demands, and sufferings, thus having limitations in providing individualized medical decisions and care. Secondly, although AI can provide accurate diagnostic and treatment suggestions, it lacks the interpersonal skills and communication abilities TCM practitioners possess. Since the doctor-patient relationship involves building mutual trust, being competent in establishing trust, understanding patient needs, and providing psychological support is very important. The renowned Tang dynasty TCM physician Sun Simiao proposed that a good doctor should have a compassionate and sympathetic heart, treat patients equally, and devote themselves wholeheartedly to curing patients’ illnesses. Patients must be listened to and soothed in the medical process, especially when facing severe diseases or emotional distress. Doctors can satisfy patients’ holistic needs by providing emotional support, understanding their demands, and offering psychological assistance, which AI currently cannot achieve. In addition, the uncertainty of individualized needs also poses a challenge. Every patient is unique with different health conditions, life backgrounds and values. Humanistic care emphasizes developing treatment plans based on patients’ personalized needs. However, AI can hardly fully consider these differences since it relies primarily on big data and pattern matching. This may lead to neglect of patients’ individualized needs and preferences, especially in conducting external therapies such as acupuncture and tuina. AI may still have gaps compared to real-world physicians, impacting the accuracy of medical decisions and patient satisfaction.

Therefore, the current research focuses on the “Patient first; Person first” research paradigm (30). This paradigm emphasizes clinical outcomes, patient-centered care, healthcare quality, and equity (31). Within this research framework, the development of artificial intelligence and the doctor-patient relationship becomes crucial for shared decision-making (6). Treatment plans and management strategies are collaboratively formulated through the active participation of both doctors and patients in the decision-making process. This paradigm emphasizes the importance of humanistic care and individualized nursing throughout healthcare. It focuses on improving healthcare outcomes and providing equal and just healthcare services. Adopting the shared decision-making model facilitated by AI can enhance communication and trust between doctors and patients, thus promoting better treatment outcomes and patient satisfaction.

Further exploration is needed to determine whether AI-assisted decision-making can reduce patient decision conflicts, improve patient knowledge levels, and enhance patient satisfaction. Artificial intelligence in the field of TCM is currently unable to achieve personalized clinical decision-making in collaboration with patients.

#### Inability of artificial intelligence to perform all diagnostic and treatment tasks

From the perspective of TCM diagnosis and treatment, it is essential to have a human TCM practitioner. Only a human TCM practitioner can understand the seven emotions of the patient as a human being. The seven emotions refer to normal emotional activities such as joy, anger, worry, contemplation, sadness, fear, and surprise, which generally do not cause illness. However, intense or prolonged emotional stimuli beyond the body’s normal capacity can lead to organ imbalances, disruption of the balance between yin and yang, and disturbances in qi and blood circulation, resulting in disease onset. The current development of AI cannot grasp human beings’ complex and advanced emotions, let alone achieve scientific TCM diagnosis through emotions. AI only enhances certain human brain functions and cannot replace human thinking. As Engels said, “We can use experimental methods to reduce ‘thinking’ to the molecular and chemical movements in the brain, but does that undoubtedly encompass the essence of thinking?” (32). Furthermore, AI-assisted decision-making can generate personalized risk predictions based on data such as electronic medical records. However, when the training data does not match the specific decision-making target population or insufficient training data, it can lead to data biases or discrimination. For example, ignoring differences among vulnerable groups may result in unfairness (33).

#### Difficulty establishing trust between humans and artificial intelligence in the field of TCM

In the TCM domain, AI faces considerable challenges in establishing trust for diagnosis and treatment. The information sources for TCM prescriptions are primarily various medical books or journals, but the collection is not comprehensive. Traditional TCM literature often contains complex and ambiguous terminology, and AI tends to lose substantial information when processing the raw data, making it difficult to obtain accurate results. More importantly, the practical experience of traditional TCM has not been fully supplemented into public databases and replicable from tradition to modernity. Unlike modern medicine, where each major discovery is added to the public knowledge system and profoundly changes evidence-based clinical practice, the ancient practical techniques and diagnostic/treatment experience of TCM have not been extensively documented and openly shared. This may relate to Chinese history’s political and cultural milieu, where authority and confidentiality were important values. Therefore, many precious TCM knowledge and skills were confined to transmission among physicians for a long period instead of being integrated into the public medical knowledge system. In addition, current research lacks standardization of the collected data, such as disease names, syndromes, formula names, historical dosage of medicines, and manifestations of efficacy. The application of modern data mining techniques remains immature and imperfect in the TCM field, with issues of bias and inaccuracy. Moreover, the TCM field lacks a unified and comprehensive terminology database (34). TCM clinical data tend to be incomplete and subjective, impacting the performance of models trained on such data (35). Data integration and lack of standardized terminology also present difficulties (36). More critically, as TCM emphasizes individualized treatment adjustment based on the specific condition, physicians must possess extensive clinical experience and a holistic grasp of the illness. If AI algorithms perform reasoning in a rigid template manner, capturing subtle differences in individual conditions would be hard, which is also a major challenge for current AI applications in TCM.

#### Legislation on AI-assisted TCM diagnosis and treatment remains incomplete

There are no clear legal definitions or provisions regarding medical disputes caused by AI-assisted TCM diagnoses (37). While AI diagnostics far surpass physicians in speed and may achieve clinical accuracy levels, responsible entities in case of errors require rational demarcation. A study (38) analyzed the applications and issues of AI in healthcare, noting the need to define accountability in medical AI development. Under Chinese laws for non-fault injury compensation, damages only cover mistakes through selective remedy, excluding complications and accidents from redress. Healthcare facilities bear no liability. Patient data from intelligent diagnostic robots also lacks legal safeguards (39). Widespread clinical adoption will generate vast personal health records amidst deficiencies in health information legislation, ambiguous data ownership, and weak public awareness of privacy rights - increasing security risks under incomplete governance frameworks. Regarding “intelligent surgery robots” or “AI-assisted TCM diagnostics,” new technologies require institutional and legal responses. Regulations in Beijing stipulate internet clinics strengthen drug oversight and prohibit automated prescriptions before patient consent. However, informed consent legislation remains deficient for AI care. Improved clinical practice guidelines and strengthened data protection could help manage these transitions involving multi-stakeholders (40).

## Discussion

Integrating TCM diagnosis and treatment with AI has drawn widespread attention in healthcare. TCM embodies the valuable experience and original thinking accumulated from thousands of years of medical practice. Meanwhile, rapid AI advancement brings new opportunities for TCM. However, the practitioner’s role in diagnosis and therapeutics remains irreplaceable, given TCM’s focus on life facets. Treatments carry risks and responsibilities that AI cannot fully assume. While AI shows vast potential in TCM, it cannot entirely substitute clinician expertise. Public trust in AI-assisted TCM still faces challenges. Establishing full human-AI confidence necessitates further research and practice. Additionally, legal definitions and regulations clarifying AI diagnostic accountability in TCM practices are needed to ensure safety and quality.

In essence, the advent of the AI era bestows upon traditional Chinese medicine (TCM) practitioners a spectrum of possibilities and trials. As a system imbued with profound expertise and distinctive cogitation, TCM can harness the potential of artificial intelligence (AI) to engender enhanced efficacy in data analysis and theoretical validation. Nevertheless, it is imperative to strike a harmonious equilibrium between technological progress and the preservation of humanistic care while advocating for the integration of AI within the realm of TCM.

## Data availability statement

The original contributions presented in the study are included in the article/supplementary material, further inquiries can be directed to the corresponding author.

## Author contributions

WL: Conceptualization, Writing – original draft, Writing – review & editing. XG: Manuscript revision, Research design. SL: Formal analysis, Investigation, Writing – review & editing. LX: Financial support, Manuscript revision. XZ: Formal analysis, Methodology, Writing – review & editing. LY: Conceptualization, Formal analysis, Writing – original draft, Writing – review & editing.
